# Supplementary material for: Bodyweight change and cognitive performance in the older population
Source: PLoS One. 2021 Apr 21;16(4):e0249651. doi: 10.1371/journal.pone.0249651 (PMC8059803; doi:10.1371/journal.pone.0249651)
Supplement: S1 Fig — (PDF) [file pone.0249651.s001.pdf]

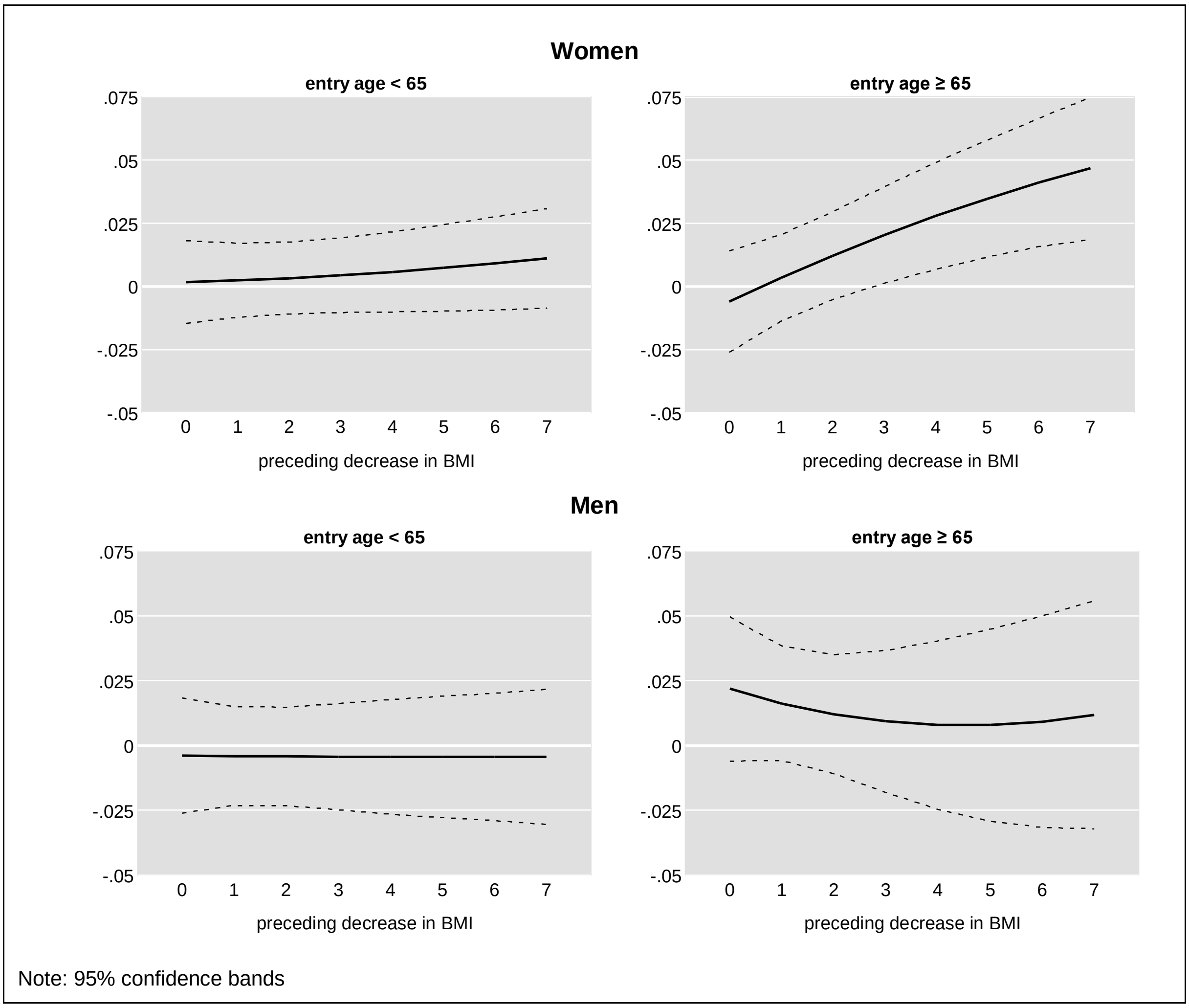

Figure A: Effects of a 1 unit BMI increase on cognition, conditional on preceding weight loss (by subsample)

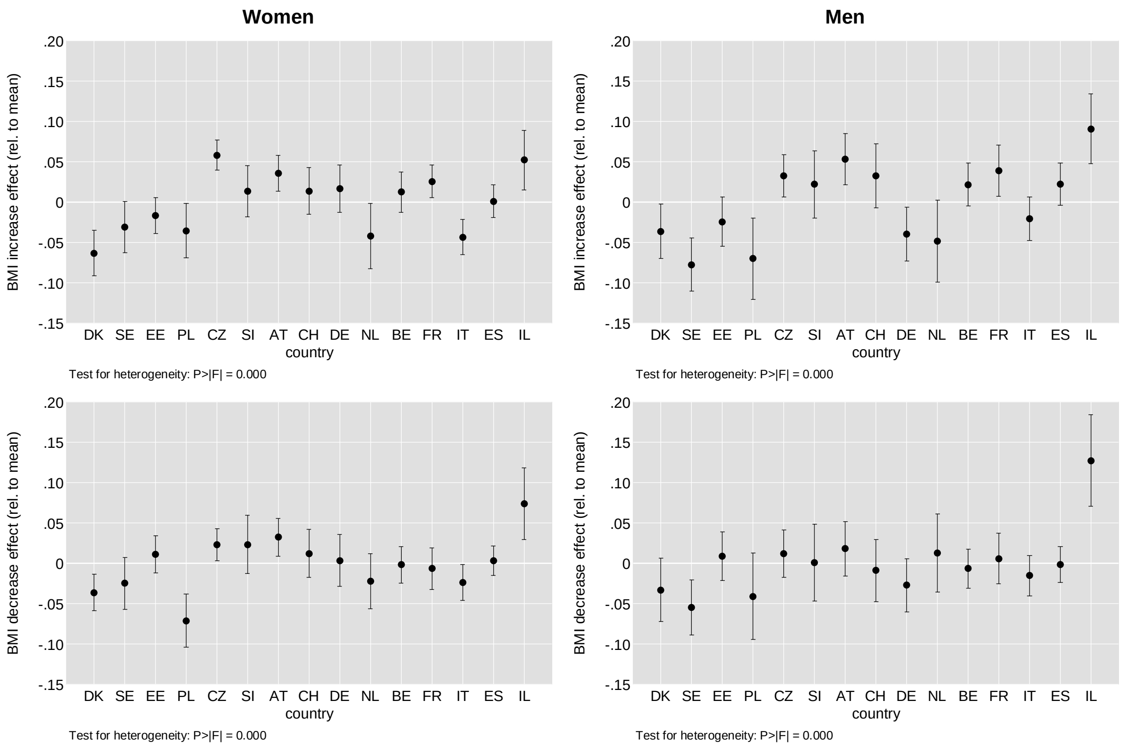

Note: 95% confidence bands

Figure B: Effect heterogeneity by country. Deviations from mean effect
