# Supplementary material for: Bodyweight change and cognitive performance in the older population
Source: PLoS One. 2021 Apr 21;16(4):e0249651. doi: 10.1371/journal.pone.0249651 (PMC8059803; doi:10.1371/journal.pone.0249651)
Supplement: S1 Table — (PDF) [file pone.0249651.s002.pdf]

Table A: BMI and cognitive performance. Regression models (unweighted)

Panel a: women	POLS	POLS (m)	BE	FE	FE-a	FE-a+	FE-aII+	FEIS
BMI	-0.015***	-0.004***	-0.005***	0.007***				
	(0.001)	(0.001)	(0.001)	(0.002)				
BMI increase					0.004	0.004	0.004	0.000
					(0.003)	(0.003)	(0.003)	(0.006)
BMI decrease					-0.011***	-0.009***	-0.007*	-0.007
					(0.003)	(0.003)	(0.003)	(0.006)
illnes-related BMI decrease							-0.008	
							(0.006)	
Age $(F_{2, 32466})$		3528.33***	3572.22***	252.91***	247.39***	214.55***	213.31***	42.93***
Education $(F_{6, 32466})$		754.66***	719.06***					
Country $(F_{14, 32466})$		203.48***	204.75***					
Grip strength $(F_{2, 32466})$						41.53***	41.15***	8.17***
Illness $(F_{6, 32466})$						29.35***	29.09***	6.46***
Physical activity $(F_{3, 32466})$						9.87***	9.81***	6.28***
N_{obs}	87777	87777	87777	87777	87777	87777	87777	59611
N_{resp}	32467	32467	32467	32467	32467	32467	32467	18204
R^2	0.005	0.321	0.434	0.011	0.011	0.017	0.017	0.006
**Panel b: men**								
BMI	-0.000	-0.000	-0.001	0.007**				
	(0.001)	(0.001)	(0.001)	(0.002)				
BMI increase					0.000	-0.001	-0.001	-0.007
					(0.004)	(0.004)	(0.004)	(0.008)
BMI decrease					-0.013***	-0.009**	-0.005	-0.018*
					(0.003)	(0.003)	(0.004)	(0.007)
illnes-related BMI decrease							-0.022**	
							(0.008)	
Age $(F_{2, 25921})$		2577.82***	2605.07***	181.36***	157.36***	138.84***	155.67***	41.01***
Education $(F_{6, 25921})$		578.48***	505.24***					
Country $(F_{14, 25921})$		82.22***	82.67***					
Grip strength $(F_{2, 25921})$						60.56***	59.34***	11.67***
Illness $(F_{6, 25921})$						17.13***	16.31***	5.45*
Physical activity $(F_{3, 25921})$						10.45***	10.06***	3.04**
N_{obs}	69203	69203	69203	69203	69203	69203	69203	45101
N_{resp}	25922	25922	25922	25922	25922	25922	25922	13700
R^2	0.000	0.251	0.353	0.010	0.010	0.018	0.019	0.008

* $p<0.05$, ** $p<0.01$, *** $p<0.001$; clustered SE in parentheses; R^2 refers to explained between variation for the BE models and within variation for FE and FEIS models; model degrees of freedom for F-tests in FEIS model reduce to 18203 (women) and 13699 (men), respectively.

Table B: BMI and cognitive performance. Regression models (weighted)

**Panel a: women**	**POLS**	**POLS (m)**	**BE**	**FE**	**FE-a**	**FE-a+**	**FE-aII+**	**FEIS**
BMI	-0.013***	-0.004***	-0.005***	0.008***				
	(0.001)	(0.001)	(0.001)	(0.002)				
BMI increase					0.004	0.005	0.005	0.006
					(0.003)	(0.003)	(0.003)	(0.006)
BMI decrease					-0.011***	-0.009**	-0.007*	-0.005
					(0.003)	(0.003)	(0.003)	(0.006)
illnes-related							-0.007	
BMI decrease							(0.006)	
Age $(F_{2, 32456})$		3116.57***	3231.06***	232.06***	224.31***	194.46***	193.59***	40.67***
Education $(F_{6, 32456})$		721.21***	690.13***					
Country $(F_{14, 32456})$		194.79***	196.68***					
Grip strength $(F_{2, 32456})$						45.94***	45.58***	9.26***
Illness $(F_{6, 32456})$						29.22***	28.96***	6.54***
Physical activity $(F_{3, 32456})$						9.22***	9.17***	5.65***
N_{obs}	87754	87754	87754	87754	87754	87754	87754	59599
N_{resp}	32457	32457	32457	32457	32457	32457	32457	18200
R^2	0.004	0.342	0.456	0.012	0.012	0.019	0.019	0.007
**Panel b: men**								
BMI	0.002	0.001	0.001	0.008**				
	(0.001)	(0.001)	(0.002)	(0.002)				
BMI increase					0.001	-0.001	-0.001	-0.004
					(0.004)	(0.004)	(0.004)	(0.008)
BMI decrease					-0.014***	-0.009**	-0.006	-0.012
					(0.003)	(0.003)	(0.004)	(0.008)
illnes-related							-0.021*	
BMI decrease							(0.009)	
Age $(F_{2, 25914})$		2406.13***	2462.53***	162.60***	162.51***	138.84***	137.42***	28.33***
Education $(F_{6, 25914})$		508.54***	496.02***					
Country $(F_{14, 25914})$		82.55***	82.99***					
Grip strength $(F_{2, 25914})$						61.18***	60.02***	12.19***
Illness $(F_{6, 25914})$						16.63***	15.88***	5.24*
Physical activity $(F_{3, 25914})$						10.36***	10.00***	3.50**
N_{obs}	69187	69187	69187	69187	69187	69187	69187	45089
N_{resp}	25915	25915	25915	25915	25915	25915	25915	13696
R^2	0.000	0.251	0.353	0.010	0.010	0.019	0.019	0.009

* p<0.05, ** p<0.01, *** p<0.001; clustered SE in parentheses; R^2 refers to explained between variation for the BE models and within variation for FE and FEIS models; model degrees of freedom for F-tests in FEIS model reduce to 18199 (women) and 13695 (men), respectively.

Table C: BMI and cognitive Performance. Regression models (subsample ≥ 65)

Panel a: women	POLS	POLS (m)	BE	FE	FE-a	FE-a+	FE-aII+	FEIS
BMI	-0.004**	0.000	-0.000	0.010***				
	(0.002)	(0.001)	(0.001)	(0.003)				
BMI increase					0.007	0.008	0.008	0.009
					(0.004)	(0.004)	(0.004)	(0.010)
BMI decrease					-0.012**	-0.009***	-0.008	-0.006
					(0.004)	(0.004)	(0.004)	(0.009)
illnes-related BMI decrease							-0.006 (0.008)	
Age ($F_{2, 14005}$)		1198.15***	893.81***	171.75***	100.43***	73.74***	67.66***	11.90***
Education ($F_{6, 14005}$)		328.08***	272.99***					
Country ($F_{14, 14005}$)		91.34***	84.11***					
Grip strength ($F_{2, 14005}$)						21.52***	18.77***	5.51**
Illness ($F_{6, 14005}$)						19.59***	16.49***	2.52*
Physical activity ($F_{3, 14005}$)						6.22***	4.83**	2.43
N_{obs}	37303	37303	37303	37303	37303	37303	37303	24637
N_{resp}	14006	14006	14006	14006	14006	14006	14006	7572
R^2	0.000	0.278	0.376	0.018	0.018	0.027	0.027	0.007
**Panel b: men**								
BMI	0.003	0.003	0.001	0.014***				
	(0.002)	(0.002)	(0.002)	(0.004)				
BMI increase					0.009	0.006	0.006	0.005
					(0.006)	(0.006)	(0.006)	(0.013)
BMI decrease					-0.017***	-0.012*	-0.006	-0.018
					(0.005)	(0.005)	(0.006)	(0.011)
illnes-related BMI decrease							-0.022** (0.008)	
Age ($F_{2, 11556}$)		798.81***	581.16***	102.31***	58.43***	32.14***	29.73***	18.66***
Education ($F_{6, 11556}$)		257.78***	219.09***					
Country ($F_{14, 11556}$)		40.34***	35.57***					
Grip strength ($F_{2, 11556}$)						35.27***	30.69***	5.68**
Illness ($F_{6, 11556}$)						12.27***	9.48***	3.09**
Physical activity ($F_{3, 11556}$)						1.73	0.74	0.79
N_{obs}	30328	30328	30328	30328	30328	30328	30328	18952
N_{resp}	11557	11557	11557	11557	11557	11557	11557	5775
R^2	0.000	0.228	0.315	0.014	0.014	0.025	0.026	0.011

* p<0.05, ** p<0.01, *** p<0.001; clustered SE in parentheses; R^2 refers to explained between variation for the BE models and within variation for FE and FEIS models; model degrees of freedom for F-tests in FEIS model reduce to 7571 (women) and 5774 (men), respectively.

Table D: BMI and cognitive performance. Regression models (subsample < 65)

Panel a: women	POLS	POLS (m)	BE	FE	FE-a	FE-a+	FE-aII+	FEIS
BMI	-0.021***	-0.007***	-0.008***	0.005*				
	(0.001)	(0.001)	(0.001)	(0.002)				
BMI increase					0.001	0.001	0.001	-0.005
					(0.004)	(0.003)	(0.003)	(0.008)
BMI decrease					-0.009*	-0.008*	-0.006	-0.006
					(0.004)	(0.004)	(0.004)	(0.007)
illnes-related BMI decrease							-0.010	
							(0.008)	
Age ($F_{2, 18460}$)		111.45***	372.27***	80.13***	67.10***	67.89***	66.06***	23.21***
Education ($F_{6, 18460}$)		426.53***	487.29***					
Country ($F_{14, 18460}$)		113.77***	131.51***					
Grip strength ($F_{2, 18460}$)						21.38***	18.81***	1.73
Illness ($F_{6, 18460}$)						10.43***	11.14***	3.52**
Physical activity ($F_{3, 18460}$)						4.36**	5.43***	3.56*
N_{obs}	50474	50474	50474	50474	50474	50474	50474	34974
N_{resp}	18461	18461	18461	18461	18461	18461	18461	10632
R^2	0.013	0.196	0.291	0.006	0.006	0.011	0.011	0.006
**Panel b: men**								
BMI	-0.009***	-0.002	-0.003	0.002				
	(0.001)	(0.001)	(0.001)	(0.003)				
BMI increase					-0.005	-0.006	-0.006	-0.014
					(0.005)	(0.005)	(0.005)	(0.010)
BMI decrease					-0.010*	-0.007	-0.005	-0.018
					(0.005)	(0.005)	(0.005)	(0.010)
illnes-related BMI decrease							-0.016	
							(0.012)	
Age ($F_{2, 14364}$)		109.67***	337.68***	77.88***	65.62***	74.52***	72.72***	16.68***
Education ($F_{6, 14364}$)		258.61***	313.39***					
Country ($F_{14, 14364}$)		45.26***	54.12***					
Grip strength ($F_{2, 14364}$)						27.53***	24.22***	6.58**
Illness ($F_{6, 14364}$)						5.18***	5.59***	2.49*
Physical activity ($F_{3, 14364}$)						12.79***	12.94***	5.89***
N_{obs}	38875	38875	38875	38875	38875	38875	38875	26149
N_{resp}	14365	14365	14365	14365	14365	14365	14365	7925
R^2	0.002	0.133	0.208	0.007	0.007	0.014	0.14	0.009

* p<0.05, ** p<0.01, *** p<0.001; clustered SE in parentheses; R^2 refers to explained between variation for the BE models and within variation for FE and FEIS models; model degrees of freedom for F-tests in FEIS model reduce to 10631 (women) and 7924 (men), respectively.

Table E: BMI and cognitive performance. Differences in estimates between the older and younger subsamples ($\beta_{\geq 65}$ - $\beta_{<65}$) as reported in tables C and D

Panel a: women	POLS	POLS (m)	BE	FE	FE-a	FE-a+	FE-aII+	FEIS
BMI	0.017***	0.008***	-0.008***	0.005				
	(0.002)	(0.002)	(0.002)	(0.004)				
BMI increase					0.006	0.006	0.006	0.014
					(0.004)	(0.005)	(0.005)	(0.012)
BMI decrease					-0.004	-0.002	-0.002	0.000
					(0.005)	(0.005)	(0.006)	(0.011)
illnes-related							0.003	
BMI decrease							(0.011)	
**Panel b: men**								
BMI	0.013***	0.005*	0.004	0.011*				
	(0.002)	(0.002)	(0.002)	(0.005)				
BMI increase					0.014	0.013	0.013	0.019
					(0.008)	(0.008)	(0.008)	(0.017)
BMI decrease					-0.008	-0.004	-0.001	0.000
					(0.007)	(0.007)	(0.007)	(0.015)
illnes-related							-0.011	
BMI decrease							(0.017)	

* p<0.05, ** p<0.01, *** p<0.001; clustered SE in parentheses.

Table F: Disentangling the confoundedness of BMI change and cognitive performance. Results based on sample split.

	β (s.e.)		Δ	Δ attributable to		
	FE-a	*FE-a+*	*overall*	*grip strength*	*diseases*	*physical activity*
**Sample with entry age ≥ 65**						
**women**						
BMI increase	0.0073<br>(0.0042)	0.0076<br>(0.0042)	-0.0003<br>(0.0005)	0.0003<br>(0.0002)	-0.0006<br>(0.0003)	0.0001<br>(0.0001)
BMI decrease	-0.0124** (0.0039)	-0.0094* (0.0038)	-0.0029*** (0.0005)	-0.0009*** (0.0002)	-0.0019*** (0.0004)	-0.0001<br>(0.0001)
**men**						
BMI increase	0.0091<br>(0.0060)	0.0064<br>(0.0060)	0.0027*** (0.0007)	0.0020*** (0.0006)	0.0009* (0.0004)	-0.0002<br>(0.0001)
BMI decrease	-0.0173*** (0.0052)	-0.0116* (0.0051)	-0.0057*** (0.0008)	-0.0034*** (0.0006)	-0.0021*** (0.0005)	-0.0002<br>(0.0001)
**Sample with entry age < 65**						
**women**						
BMI increase	0.0012<br>(0.0035)	0.0014<br>(0.0035)	-0.0002<br>(0.0003)	0.0003<br>(0.0002)	-0.0002<br>(0.0002)	-0.0004** (0.0001)
BMI decrease	-0.0087* (0.0035)	-0.0075* (0.0035)	-0.0012*** (0.0003)	-0.0008*** (0.0002)	-0.0004* (0.0002)	0.0000<br>(0.0001)
**men**						
BMI increase	-0.0053<br>(0.0046)	-0.0063<br>(0.0046)	0.0010* (0.0005)	0.0014*** (0.0004)	0.0000<br>(0.0002)	-0.0004<br>(0.0002)
BMI decrease	-0.0098* (0.0045)	-0.0072<br>(0.0045)	-0.0026*** (0.0006)	-0.0019*** (0.0005)	-0.0007** (0.0003)	0.0001<br>(0.0002)

Note: * p<0.05, ** p<0.01, *** p<0.001; clustered SE in parentheses.
